# Health Equity and Policy Considerations for Pediatric and Adult Congenital Heart Disease Care among Minoritized Populations in the United States

**DOI:** 10.3390/jcdd11020036

**Published:** 2024-01-25

**Authors:** Keila N. Lopez, Kiona Y. Allen, Carissa M. Baker-Smith, Katia Bravo-Jaimes, Joseph Burns, Bianca Cherestal, Jason F. Deen, Brittany K. Hills, Jennifer H. Huang, Ramiro W. Lizano Santamaria, Carlos A. Lodeiro, Valentina Melo, Jasmine S. Moreno, Flora Nuñez Gallegos, Harris Onugha, Tony A. Pastor, Michelle C. Wallace, Deidra A. Ansah

**Affiliations:** 1 Texas Children’s Hospital, Department of Pediatrics, Division of Pediatric Cardiology, Baylor College of Medicine, Houston, TX 77030, USA; joseph.burns@bcm.edu (J.B.); carlos.lodeirojordan@bcm.edu (C.A.L.); jasmine.moreno@bcm.edu (J.S.M.); deidra.ansah@bcm.edu (D.A.A.); 2 Ann & Robert H. Lurie Children’s Hospital of Chicago, Northwestern University Feinberg School of Medicine, Chicago, IL 60611, USA; kallen@luriechildrens.org; 3 Center for Cardiovascular Research and Innovation, Nemours Cardiac Center, Nemours Children’s Health, Wilmington, DE 19803, USA; carissa.baker-smith@nemours.org; 4 Department of Cardiovascular Medicine, Mayo Clinic Florida, Jacksonville, FL 32224, USA; bravo.katia@mayo.edu; 5 Ward Family Heart Center, Children’s Mercy Kansas City, Kansas City, MO 64108, USA; bpcherestal.md@gmail.com; 6 Department of Pediatrics and Medicine, University of Washington, Seattle, WA 98105, USA; jason.deen@seattlechildrens.org; 7 Division of Pediatric Cardiology, UT Southwestern, Children’s Health, Dallas, TX 75390, USA; brittney.hills@utsouthwestern.edu; 8 Doernbecher Children’s Hospital, Oregon Health and Science University, Portland, OR 97239, USA; huangje@ohsu.edu; 9 Cardiac Center, Children’s Hospital of Philadelphia, Philadelphia, PA 19104, USA; lizanor@chop.edu; 10 Texas Children’s Hospital, Department of Pediatrics, Baylor College of Medicine, Houston, TX 77030, USA; valentina.melo@bcm.edu (V.M.); harris.onugha@bcm.edu (H.O.); 11 Department of Pediatrics, University of California San Francisco Benioff Children’s Hospital, San Francisco, CA 94158, USA; flora.nunezgallegos@ucsf.edu; 12 Division of Pediatric Cardiology, Yale School of Medicine, Yale New Haven Hospital, New Haven, CT 06510, USA; tony.pastor@yale.edu; 13 Department of Pediatrics, Emory University School of Medicine and Children’s Healthcare of Atlanta, Atlanta, GA 30322, USA; wallacem@kidsheart.com

**Keywords:** health equity, congenital heart disease, structural racism, health policy, underrepresented minority in medicine

## Abstract

Achieving health equity in populations with congenital heart disease (CHD) requires recognizing existing disparities throughout the lifespan that negatively and disproportionately impact specific groups of individuals. These disparities occur at individual, institutional, or system levels and often result in increased morbidity and mortality for marginalized or racially minoritized populations (population subgroups (e.g., ethnic, racial, social, religious) with differential power compared to those deemed to hold the majority power in the population). Creating actionable strategies and solutions to address these health disparities in patients with CHD requires critically examining multilevel factors and health policies that continue to drive health inequities, including varying social determinants of health (SDOH), systemic inequities, and structural racism. In this comprehensive review article, we focus on health equity solutions and health policy considerations for minoritized and marginalized populations with CHD throughout their lifespan in the United States. We review unique challenges that these populations may face and strategies for mitigating disparities in lifelong CHD care. We assess ways to deliver culturally competent CHD care and to help lower-health-literacy populations navigate CHD care. Finally, we review system-level health policies that impact reimbursement and research funding, as well as institutional policies that impact leadership diversity and representation in the workforce.

## 1. Introduction

### 1.1. Structural Racism Impacts the Early Life of CHD Populations in the U.S.

Absolute health outcomes and differences in care between groups are a result of long-standing systemic inequities and persistent structural racial-political, legal, economic, health, school, and criminal justice systems that uphold racial disparities [1]. Upstream systemic racism often accounts for downstream differences in socioeconomic position and for “the high burden of illness responsible for appalling premature loss of life arising in large part because of the conditions in which people are born, grow, live, work, and age” [2]. Due to the embeddedness of its effects in society, systemic racism is often “assumed to be the inevitable order of things” [3]. Evidence of these systemic inequities is notable, starting in fetal life in populations with CHD. While the prevalence of CHD is slightly higher among non-Hispanic White individuals compared to non-Hispanic Black and Hispanic/Latino persons [4], the technology used for improving immediate and long-term outcomes, such as prenatal detection, are less commonly employed within low socioeconomic and racially minoritized communities [5]. Lower rates of prenatal detection of conditions such as transposition of the great arteries (TGA) and hypoplastic left heart syndrome (HLHS) have been reported among Hispanic and rurally residing infants [5]. Lower rates of prenatal screening contribute to poorer outcomes among lower-socioeconomic-status (SES) and racially minoritized populations [6]. Further, differences in one-year post-surgical outcomes among infants with HLHS and TGA were more fully explained by differences in insurance status and maternal education rather than by maternal ethnicity [7].

### 1.2. Lifelong Poorer SDOH in CHD Populations Stem from Upstream Systemic Racism

Systemic racism leads to disinvestment in largely poor and predominantly marginalized and minoritized communities in the United States. The intersection between SDOH and disparities by race and ethnicity is rooted in structural racism that results in uneven access to quality schools, good-paying jobs, higher incomes, wealth accumulation, better neighborhoods, health insurance, and quality medical care [8]. Within the CHD population, these associations are notable: mortality outcomes are 1.8 times higher among non-Hispanic Black vs. White infants with HLHS, and most of the association is explained by “unmeasured factors or potentially structural racism” [9]. A less privileged socioeconomic position and lower maternal education explain a large portion of the 1-year death disparity in non-Hispanic Black and Hispanic infants with CHD [10]. These effects persist into adulthood, with disparities in mortality persisting for non-Hispanic Blacks versus non-Hispanic Whites until at least the age of 35 years [11]. Practices and policies are needed to address these health inequities to achieve healthy equity in CHD outcomes [12]. Of note, refugee populations as well as asylum seekers also have noted poorer SDOH and face significant challenges in obtaining CHD care. These vulnerable populations require that collaborative efforts be made between physicians and support services to provide assistance, and given the unique and varied needs of individual communities [13], this issue will not be further expounded upon in this document.

### 1.3. Lack of Adequate Reimbursement for Pediatric Care and Access to CHD Specialty Services for Lower SES Patients

Different reimbursement rates for pediatric vs. adult cardiology care are a source of disparity. In the U.S., pediatric healthcare services are reimbursed at a lower rate than adult healthcare services, resulting in pediatric unit closures and subsequent bed shortages. In particular, the American Hospital Association reports that between 2002 and 2011, more than 2300 pediatric beds in approximately 5000 hospitals were closed [14]. Meanwhile, free-standing children’s hospitals are operating at capacity and do not always have the bed space required to accept transfers and to care for children with complex CHD [14].

Health insurance coverage and differing patterns in health system coverage acceptance are also sources of disparity. Medicaid serves as the base of coverage for the nation’s low-income children, covering 36.8 million children in 2015. The Children’s Health Insurance Program (CHIP) complements Medicaid by covering uninsured children above Medicaid eligibility limits [15]. During the COVID-19 pandemic, there was a significant increase in pandemic-related healthcare coverage, with 5 million more children receiving Medicaid and CHIP (over 41 million in total) [16]. However, since the lapse of pandemic-related healthcare coverage in April 2023, at least 2 million children have lost health insurance coverage [17]. This loss of coverage is worse in the 10 states that did not expand Medicaid, such as Texas, where almost 60% of people who have lost coverage since 1 April 2023 are kids. Worse still is that many young adults who lose Medicaid coverage, such as those with CHD, will not have, be eligible for, or be able to afford other insurance options. Despite a recent Health and Human Services report showing that the national uninsured rate reached an all-time low in 2023 after the record-breaking Affordable Care Act (ACA)-promoted enrollment, many hospitals specializing in CHD surgeries and care do not accept ACA insurance [18]. This poses a significant problem in accessing the needed expertise required to care for complex aging CHD populations.

### 1.4. Lack of Representation of Underrepresented Minorities in Medicine

Underrepresentation of minoritized populations in medicine is a complex issue that originates from a history of structural racism. These structural barriers promote disparities at multiple levels, which effectively preclude many minority students from pursuing a career in medicine. Consequently, minority representation in medicine is significantly reduced, impacting all aspects of the practice of medicine, including patient care, research, and policy, among others. Data from the 2022 U.S. census reveal that while 19% of the population identifies as Hispanic, 14% as Black or African American, and 1.3% as Native American or Alaskan Native [19], only 5.8% of all practicing physicians are Hispanic, 5% are non-Hispanic Black, and 0.3% are Native American [20]. Between 1997 and 2017, the relative percentage of American Indian/Alaska Native (AI/AN) students decreased relative to the size of the population [21]. Further, data show that to correct the deficit of Hispanic and Black physicians starting from 2015, it would take 92 and 66 years of sustained doubling of Hispanic and Black medical students, respectively [22].

Pediatric cardiology is no exception to this lack of representation. While there is limited literature about the specific demographics of the pediatric cardiology workforce in the U.S., a recent study by Balasubramanian et al. reported that in academic pediatric cardiology programs, only 14% of the trainees and 10% of the faculty belong to racial/ethnic groups that are Underrepresented in Medicine (URiM), with very few in leadership roles [23]. There has been only a modest increase in the number of URiM physicians training in pediatric cardiology, which highlights the importance of analyzing program recruitment efforts to ensure that physicians and healthcare workers truly represent the demographics of the patients with CHD we serve [24].

## 2. System-Level Health Policy Solutions to Improve Health Equity for Minoritized Populations with CHD

### 2.1. Native American/Alaskan Native Populations

The life expectancy for American Indian/Alaska Native (AI/AN) populations in the United States is the lowest among those of any racial or ethnic group, at 65.2 years [25]. AI/AN individuals are more likely to have early-onset cardiovascular disease (CVD), with a higher rate of morbidity and mortality [26]. In fact, heart disease is the leading cause of death in AI/AN communities (19.2%), with risk factors beginning in childhood [25,27]. AI children also demonstrate the highest rate of metabolic syndrome when compared to children of any race or ethnicity in the U.S. (24.9%) [28]. Evaluations from the Strong Heart Family Study (SHFS), the largest longitudinal study of AI cardiometabolic disease, found that (1) AI youth were disproportionately obese, which is associated with hypertension and insulin resistance later in life [26]; (2) AI youth demonstrated an association between obesity and left ventricular hypertrophy with impaired cardiac function [29].

These AI/AN inequities are deeply entangled with the history of settler colonialism and historical trauma in the United States [27,30]. This structural racism continues to perpetuate health inequities, including those regarding access to care, federal funding for healthcare, land rights and allocation, and the consequences of violence, including adverse childhood experiences, the Missing and Murdered Indigenous People (MMIP) crisis, and the boarding school system [31,32]. These upstream factors, which have led to poorer SDOH and cardiovascular health in AI/AN populations, put populations with CHD from these communities at higher risk for morbidity and mortality, particularly as they age, as adult CHD populations are also at an increased risk for acquired cardiovascular disease [33].

*Potential solutions:* Efforts to mitigate the impacts of these upstream factors on CHD populations must be made at multiple levels, including national, state, and local levels. AI individuals access care in multiple settings, including Indian Health Service (IHS) facilities, Urban Indian Health Centers, tribal health facilities, and all other care delivery modes. Both the IHS and the Urban Indian Health Centers receive U.S. federal funding. Thus, federal-level advocacy efforts to improve monetary allocations may increase the availability of CHD services through these avenues. Further, a policy to encourage Indigenous sovereignty, thereby increasing the capacity of tribal governments to make financial and structural decisions to promote the health of their communities, is paramount. 

Programming efforts centered on strengths have demonstrated effectiveness in Indigenous communities. Preserving culture, language, and community all play an essential role in safeguarding AI/AN values and health. Thus, efforts that support the funding and structure of programs, including home visitation programs (e.g., the Family Spirit Home Visiting Program at the Center for Indigenous Health at Johns Hopkins University), play an important role in preventing early-onset cardiovascular disease. Further, policies that promote institutional partnerships with tribal communities are vital to understanding community needs and developing culturally sensitive programs to address these areas. 

### 2.2. Non-Hispanic Black Populations

The non-Hispanic Black population experiences both similar and unique barriers to equitable care as other minoritized groups. Racism is a core thread throughout these barriers but also offers insight into some solutions. Neighborhood deprivation and housing quality are important determinants of the cardiovascular health status, and non-Hispanic Blacks are more likely to live in neighborhoods with concentrated poverty due to the long history of racial segregation (redlining) in the U.S. [34]. Higher neighborhood-level deprivation is associated with 61% greater odds of hypertension and obesity in youth, more premature births, and worse transplant-free survival in the first decade of life following the Norwood operation [35,36].

*Potential solutions:* There is a need for policies aimed at allocating resources toward improving neighborhood-level risk factors and increasing access to medical and psychological treatment for youth to reduce the burden of CVD. 

Approximately 80% of CVD is preventable through lifestyle modifications; however, poorer SDOH can cause barriers to these changes [37]. Psychosocial stressors secondary to aspects of the physical environment (i.e., food access and neighborhood safety) contribute to increased CVD in non-Hispanic Black patients [38].

*Potential solutions:* Food assistance programs can ensure food equity through improved program coordination, increasing outreach, and simplified enrollment processes [39]. It will take efforts at multiple levels to address these barriers, but improving transportation infrastructure, providing safe exercise locations, and requiring diversity in CVD clinical studies are potential solutions [37].

Personal health literacy is the degree to which individuals can find, understand, and use information and services to inform health-related decisions and actions. Limited health literacy is prevalent among non-Hispanic Blacks, affecting 57% of this demographic [40]. Data have documented the association of low health literacy with poor health outcomes, including patients with CHD. Poor understanding of CHD in children will likely result in higher rates of unintended hospital admissions, increased resource utilization, and lower health-related quality of life. 

*Potential solutions:* We must address the role of institutions in identifying poorer individual health literacy and in improving organizational health literacy (the degree to which organizations equitably enable individuals to find, understand, and use information and services to inform health-related decisions) [41]. In 2020, part of the U.S. government’s “Healthy People 2030” initiative included “organizational health literacy” and emphasized the relationship between health literacy and health equity [42]. 

Access to health insurance and reliable provider coverage remains a concern for the non-Hispanic Black population. Despite non-Hispanic Blacks making up about 13% of the population, they represent about 25% of those uninsured. While the ACA reduced the uninsured rates of non-Hispanic Blacks, lack of insurance under the ACA persists in states that have not expanded Medicaid (many of which have large non-Hispanic Black populations).

*Potential solutions:* It is imperative to advocate for all states to expand their Medicaid coverage, as CHD patients live in all 50 states [43]. Beyond insurance, new state and federal policies that directly reverse the impact of longstanding systemic racism are needed, with the only sustainable path forward being one that concurrently targets housing, education, healthcare, economic empowerment, the built environment, and access to healthy foods [44].

### 2.3. Hispanic/Latino Populations

The Hispanic/Latino population in the U.S. faces multiple barriers to accessing quality CHD care. These barriers have led to higher rates of obesity, hypertension, increased perioperative mortality, worse surgical outcomes, and increased cardiac complications in this population compared to other ethnic groups [7,45,46,47]. Although multiple factors contribute to these disparities, three of the most impactful ones include access to health insurance, immigration status, and language barriers.

According to a recent government census report, the Hispanic/Latino population had among the highest uninsured rates in the nation, at 17.7% [48]. This is partly a result of Hispanic/Latinos often being employed in industries that are less likely to offer employer-based health coverage. The lack of insurance has a direct effect on the health of patients with CHD, as described in the study by Nuñez et al., where uninsured infants with critical CHD were found to have threefold increased mortality compared to privately insured children [49]. Undocumented immigrants and children in households of mixed citizenship status are restricted in or fear seeking out government-funded health insurance [50,51]. These factors reduce the access to frequent specialized care, which is paramount in the CHD population, and add to the financial burden on families to cover such care. Moreover, Hispanic/Latino individuals often face challenges in accessing care due to language and cultural barriers [52,53,54], which often leads to poorer communication, discrimination, lower health literacy, worse patient satisfaction, and greater utilization of emergency resources [55,56].

*Potential solutions:* It is imperative to implement strategies that could improve health equity for Hispanic/Latino patients with CHD. One approach includes expanding Medicaid to children irrespective of their immigration status. After California’s 2016 expansion to cover low-income children insurance regardless of their immigration status, there was a 34% decline in the rate of uninsured individuals who were non-U.S. citizens [57]. For those with chronic medical conditions like CHD, this decline in uninsured rates could mean lower utilization of emergency healthcare, lower likelihood of requiring urgent cardiac intervention, higher chance of keeping specialist follow-up appointments, and overall decreased morbidity and mortality [58,59]. Similarly, advocating for policies promoting a pathway to citizenship, especially for children with chronic medical needs, and combating misinformation surrounding the use of public funds and future impacts on citizenship status can increase access to social resources and promote continued access to care [57,60]. Lastly, in the context of increased telehealth capabilities, including comprehensive language interpretation, family educational resources in Spanish, and links to family advocacy groups of similar cultural and language backgrounds is crucial. Altogether, these efforts can promote health literacy, encourage community partnerships, and increase social support to improve the overall health of the Latino population with CHD.

### 2.4. Lesbian, Gay, Bisexual, Transgender, and Queer (LGBTQ) Populations

Sexual and gender minorities account for ~7.2% of self-identified adults and 19.7% of Generation Z, according to Gallup’s latest update in 2023 [61]. Still, members of the LGBTQ community continue to face significant health disparities starting in childhood and continuing through their lifetime, as compared to their cisgender peers [62]. These disparities include a wide range of health disorders, including substance abuse, sexually transmitted diseases, cancers, cardiovascular disease, obesity, and various mental health disorders [63]. Concerning cardiovascular disease, specifically, lesbian, gay, and bisexual adults were 36% less likely than heterosexual adults to have ideal heart health, and transgender and gender-diverse individuals also had increased rates of cardiovascular disease as compared to cisgender individuals. Known factors that influence these poor outcomes in this population include minority stress, legal discrimination, victimization, familial rejection, lower SES, healthcare discrimination, healthcare avoidance, and intersectionality (the complex, cumulative way in which the effects of multiple forms of discrimination (such as racism, sexism, and classism) combine, overlap, or intersect, especially in the experiences of marginalized individuals or groups) [64]. Many individuals are reluctant to share their sexual orientation or gender identity with HCPs out of fear of misunderstanding or maltreatment or simply out of wanting to avoid making the provider feel uncomfortable [65].

*Potential solutions:* While these factors are challenging to ameliorate from a policy level alone, health policies that target these issues can decrease the impact of many of these risk factors. These policies include those that prohibit health insurance discrimination based on sexual orientation and gender identity (laws currently in 14 states), provide legal protection in employment, housing, and medical care for LGBTQ individuals, and require coverage of gender-affirming care. While many LGBTQ individuals are very adept at code switching to blend in with cis-gender heterosexual people, CHD providers have a unique opportunity, given the regularity of clinic visits and the long-term therapeutic relationships, to intervene in specific ways that can help LGBTQ patients live a healthier life. Content on LGBTQ health should be integrated into health professions curricula and continuing education for practicing clinicians.

In addition, policies that direct resources to research on specific factors associated with poor health in this understudied population can help direct future initiatives to improve health outcomes. There is a need to develop and test interventions that address multilevel stressors that affect the cardiovascular health of LGBTQ adults. Expanding the mechanistic knowledge about how LGBTQ-specific minority stressors affect the cardiovascular health of LGBTQ adults is crucial to developing and tailoring multilevel cardiovascular health interventions for this population [66]. Engagement with preventive care is significantly lower in sexual and gender minority women in comparison to heterosexual individuals, which can be due to both patient and healthcare provider factors. There are simple steps that CHD providers can take to provide a safe environment for LGBTQ patients. Avoiding assumptions when asking about romantic relationships, sexual behavior, and plans for having children and adopting a gender-neutral language can allow individuals to express a preference for their pronouns, discuss their gender identity, or disclose their sexual orientation [65]. An improved medical education curriculum across the spectrum of medical training (from pediatric to adult) is necessary to provide not only culturally competent care but also specialized care for sexual and, particularly, gender-diverse individuals.

### 2.5. Persons with Disabilities Living with CHD

Individuals with CHD are more susceptible to disabilities throughout their lifespan. These disabilities may include difficulties with hearing, vision, cognition, mobility, self-care, and the ability to live independently [67]. Data show that 25% of the CHD population have extracardiac structural malformations [68]. In childhood, those with CHD have a twofold higher risk for developmental disability, even if they have simple CHD [69]. Data also show that two in every five adults with CHD may have disabilities, and these are associated with impaired health-related quality of life [67]. In fact, the prevalence of disability types is five to eight times higher in adults with CHD than in the general population [67]. Even more concerning are how disabilities disproportionately impact other existing vulnerable communities. For example, individuals with CHD who have at least one disability have a greater odds of being non-Hispanic Black [67], and compared to patients with a higher SES, those with a lower SES were noted to have more disabilities [70].

*Potential solutions:* When assessing and interacting with CHD populations, particularly when they have physical disabilities or are from vulnerable communities, physicians need to be mindful of intersectionality and the higher potential for cognitive disabilities, as well as of the potential impact that this has on quality of life. Physicians also need to (1) be aware of barriers that people with disabilities face when accessing health care services; (2) promote the inclusion of persons with disabilities in ancillary therapy programs (physical therapy, cognitive therapies, etc.); (3) emphasize long-term disease prevention and health promotion programs; and (4) underscore potential qualifications for insurance coverage through social security or Medicaid in this vulnerable population.

### 2.6. Reimbursement and Research Funding

The current healthcare reimbursement model in the U.S. promotes disparities for minoritized children and adults with disabilities. Medicaid and Medicare are the largest public health insurance programs for children and adults, respectively. A larger proportion of minoritized children are covered by Medicaid, whose reimbursement rates average only 70% of Medicare rates [71]. The disparities affecting minoritized patients with public insurance widen with the variation of the Medicaid reimbursement rates for the same services across states and the variability in healthcare systems’ acceptance of Medicaid and Medicare plans. 

Restrictive insurance policies also promote inadequate access to care for patients with CHD, especially those living in remote geographic areas. Telemedicine is a powerful tool to promote equity and access to care. However, its use is constrained by low reimbursement rates and the inability to cross state lines [72]. In addition, vital ancillary services such as nutrition, mental health, neurodevelopmental, and transition medicine services struggle to be sustainable. These services are largely under-reimbursed or not covered by health insurance, despite making clear positive impacts on patient outcomes. 

*Potential solutions:* Health insurance coverage is particularly important for minoritized patients with CHD [73]; thus, expanding Medicaid enrollment is critical to closing the healthcare access gap and improving access to quality care. This type of coverage would benefit patients with CHD by providing them with automatic enrollment upon birth, increasing eligibility and coverage up to the age of 26, and achieving Medicare parity pay and equitable reimbursement and portability across state lines, as not all states or geographic locations have specialized CHD providers or heart centers [74].

The National Institutes of Health (NIH) has increasingly funded research projects for congenital heart disease (CHD) over the last decade. From 2005 to 2015, USD 991 million was awarded to 633 projects, mainly in basic or translational research focused on cardiac developmental biology [75]. In 2018, the Congenital Heart Futures Reauthorization Act was signed into law to enhance research and surveillance efforts at the Centers for Disease Control and Prevention and the NIH, specifically toward studying CHD across the lifespan [76]. Increased efforts towards capitalizing on the momentum gained by this law are needed, as this reauthorization will expire in 2024. Further, research funding opportunities should increase given CHD cost and resource utilization in all areas (basic, translational, clinical, qualitative) and across the lifespan.

## 3. Institutional- and Organizational-Level Health Policies to Improve Health Equity for Minoritized Populations with CHD in the United States

### 3.1. Representation/Recruitment/Retention of Underrepresented Minorities in Pediatric Cardiology

Despite efforts to diversify medical school enrollment, the American Association of Medical Colleges for the 2023–2024 year reported that matriculation in medical school was only 0.15% for American Indians or Alaska Natives (AIs/ANs), 8% for non-Hispanic Blacks, and 6.5% for Hispanics [77]. The data are even more alarming for pediatric cardiology, where <8% of physicians are underrepresented in medicine (URiM). Considering that non-White minorities currently comprise roughly 50% of all children in the U.S., these numbers expose the significant disparity that exists between the U.S. population and the U.S. healthcare workforce, a known contributor to health inequity [12].

Contributing factors to the lack of URiM individuals in the healthcare field include the cost of education, the insufficient access to tools for academic preparation for medical school admission requirements, the lack of racially or ethnically concordant mentors, the limited exposure to health careers, poor advising, and an implicit bias in the admission process [78]. Data show that physician concordance by race, culture, and language increases the quality of care, patient satisfaction and adherence, and the likelihood of patients participating in preventative care [12]. URiM physicians are significantly more likely to care for minority, publicly insured, and uninsured patients and to conduct research to inform health policy on issues affecting these populations [12]. Many URiM individuals are not exposed to pediatric subspecialty care (such as pediatric cardiology) during their medical school training, and thus, this further reduces the likelihood of diversifying pediatric cardiology. Therefore, it is of utmost importance to establish policies and strategies at the institutional level that will increase URiM presence in academic, hospital, and subspecialty pediatric programs and leadership positions in these institutions. 

*Potential solutions:* The first solution is to acknowledge the history of systemic racism within medicine and develop programs and strategies that rectify past inequitable recruitment and subsequent training [79]. This may be accomplished by creating and endowing high school and undergraduate pathway programs, supporting policies to secure positions for URiM students, and providing mentorship and sponsorship [21,78]. Another strategy involves alleviating the financial burden of medical education in the U.S. by providing funding for students who attend institutions that primarily serve minority populations, including Tribal Colleges and Universities, Hispanic-Serving Institutions, and Historically Black Colleges and Universities. Medical school admission processes and recruitment to residency and fellowship programs should also be reassessed and recalibrated, advocating for a more holistic process in which skills such as language, cultural competence, overcoming adversity, and representation are valued. Institutions must also address the workplace culture, build promotional structures that encourage diversity to retain and promote URiM academic physicians, and support faculty in scholarly and service work related to diversity and health equity [79,80]. Developing and supporting a diverse physician workforce is imperative to improving patient health outcomes. Beyond this, diversifying the nursing workforce and ancillary workforce (physical therapy, child life, etc.) is also critically important to help address systemic racism in healthcare, as well as to ensure that they reflect the communities that they are serving. 

### 3.2. Cultural Considerations in CHD Care Delivery

Organizations must provide training in cross-cultural care and associated communication skills to provide equitable and effective care, increase diversity in research participation, improve research generalizability, and move toward equitable outcomes. The Office of Minority Health recommends this standard of care, adopted as an accreditation requirement by the Joint Commission and supported by the cardiology research community [81,82,83]. Further, data suggest that patient satisfaction with care and adherence increases when providers are viewed as having both cultural competence (the ability to engage knowledgeably with people across cultures) and cultural humility (understanding the complexity of identities and the lifelong process of never being fully competent about the evolving and dynamic nature of a patient’s experiences) [84,85,86]. As patients’ culture influences their lived experience and outcomes, training health professionals in the approach to patients with multiple sociocultural identities is imperative.

*Potential solutions:* Cross-cultural training has traditionally focused on race and ethnicity. However, this training should also extend to other contributors of intersectionality such as socioeconomic background, religious or spiritual practice, disability, and sexual or gender identity [12]. 

Training in institutions has typically focused on cultural competency. However, competency falsely suggests that culture can be mastered. Instead, cultural humility should be promoted, which acknowledges that there is no finite knowledge base related to the provision of care for diverse populations. In contrast, with ongoing self-introspection, lifelong commitment to learning, and intentional efforts to deconstruct paternalistic medical frameworks, transition to an effective culture of patient advocacy can occur [87]. 

While guidance for cultural humility training in clinical cardiology does not exist, the Joint Commission and the literature offer suggestions on approaching it [81,88,89,90]. Topics covered include implicit bias, recognition of privilege, communication with patients from diverse backgrounds, and principles to address social determinants of health [91,92]. Training formats include discussion, online modules, videos, interaction with standardized patients, and cultural immersion. Program effectiveness has been measured through surveys, debriefing, or journaling [93]. While data have not investigated if cultural humility training directly impacts patient outcomes, trained providers report improved comfort with caring for diverse patients and increased awareness of their biases and attitudes that may affect care [88,89,90,94,95]. Providers participating in immersion programs have also enhanced sociocultural skills related to the patient–doctor relationship and communication [96,97]. This and increasing the diversity of medical providers and researchers can work synergistically to increase participation in research initiatives. 

To build trust with marginalized communities and strengthen clinical care, the impact of culture on patient attitudes and on the approach to care should be recognized and prioritized.

### 3.3. Navigation of a Complex Medical System

Minoritized populations in the U.S. face significant disparities in access to healthcare and the quality of care received. Systemic issues within the healthcare system often perpetuate these disparities, including institutional- and organizational-level policies that create barriers to care. For minoritized populations with CHD, who often need lifelong care, health policies must be developed to aid in addressing these disparities. 

*Potential solutions:* It is imperative to give all populations the necessary tools to navigate this complex system regardless of language or the health literacy level. Several studies investigated the utility of patient navigators in several different medical contexts. Many studies have shown improved patient outcomes, ranging from fewer readmissions to greater access to standard health services [98,99,100,101,102]. Further, hospitals must invest in interpreter services to shorten the lengths of stay, avoid adverse events, and improve patient communication and confidence in their care [103,104,105,106,107,108]. Hospitals must also engage in specific care metrics during an inpatient stay, such as target-based care, to reduce outcome variation, including the length of stay, for all patients. Using this framework to eliminate health disparities by highlighting when a patient’s course is not meeting specific targets may move the health equity needle [109].

Navigating access to care throughout the lifespan is paramount to equitable outcomes. It is essential to delineate inequities in the transition of pediatric to adult CHD care, as this is the highest risk period for falling out of care, and lapses in care are predictors for increased morbidity and poor long-term CHD outcomes, particularly for minority populations. SDOH, such as poverty, lack of private insurance, difficulties in housing, low level of parental education, being an immigrant, being a minoritized population, shortage of food supply, and transportation barriers, are linked to several adverse clinical outcomes, including missed appointments and loss to follow-up [110,111]. Fewer than 30% of adolescents with CHD successfully transition to adult care; this percentage is even lower for minority and lower-socioeconomic-status populations [112]. Finally, having the ability to access quality congenital cardiac care across the lifespan is critical. Families living in rural communities are disproportionately burdened by increased travel time to receive appropriate levels of care [113]. Institutions and healthcare organizations must account for how changes in their practice policies can further drive or bridge disparities in care delivery, such as providing satellite clinics or telemedicine access to patients. 

### 3.4. Leadership and Representation at Institutions, National Organizations, and Editorial Boards

Despite the benefits of promoting diversity in medicine [114], pediatrics continues to lag, as underrepresented minorities comprise only 16% of the pediatric residents, with the percentage of underrepresented physicians in the field increased by only 2% in the last decade [24]. Furthermore, disparities have also been observed in medical faculty appointments, section chiefs, and journal editorial boards of other medical specialties [115].

*Potential solutions:* There is an urgent need to address the disparities preventing health equity in those leading medicine: data show that women achieve lower academic ranks versus men, and among URiM groups, women comprise the majority of assistant professors and instructors, which suggests that the intersectionality of sex and racial and ethnic group may further disadvantage this group relative to their male counterparts, particularly with regard to attaining higher rank and leadership positions [116]. Beyond this, there is a need for an increased focus on the retention of URiM individuals in academic medicine, as there is currently an epidemic of these individuals leaving academic medicine [117]. Institutional support of (1) diverse physician-scientists who focus on research, education, and interventions to reduce health inequities and (2) pathways for promotion based on health equity work may improve URiM physician retention. Further, all fields in medicine, including pediatric cardiology, have URiM physicians that shoulder the burden of a minority tax (the burden of extra responsibilities placed on minority faculty in the name of diversity), which often is not recognized as a contributor to physician burnout and lack of URiM retention in academic medicine [118]. Finding ways to have those extra responsibilities be compensated or count toward promotion would surely help. 

Large society conferences and participation in national organizations offer a unique opportunity to showcase the contributions of underrepresented individuals and introduce new perspectives to the broader scientific audience by fostering interactions between individuals with diverse backgrounds, race/ethnicity, gender, specialties, and worldviews. The impact is attenuated, however, if diverse speakers are only considered for workshops focused on diversity, equity, and inclusion rather than given opportunities to showcase their unique scientific contributions. Women and certain minority groups remain underrepresented as invited speakers out of proportion to their underrepresentation in the medical field, which may further compromise their professional development [119]. 

*Potential solutions:* Improving diversity in speakership at national conferences, particularly on the main stage, requires intention on the part of the society. This begins with intentional diversification of the program and meeting committees and an ongoing outspoken commitment from the society’s national leadership to promoting speaker diversity [120].

Eliminating racial bias in science cannot be achieved without establishing diverse editorial boards. The lived experience cannot be replicated or fabricated, and the notion that the pervasive acceptance of racial inferiority can be eliminated from scientific journals in the setting of a monolith editorial board has proven challenging. URiM representation on editorial boards and in leadership roles within journals, particularly in cardiology, is scarce [121].

*Potential solutions:* Greater editorial board diversity is good not only for scholars but also for readers. Furthermore, greater diversity in the editorial board of high-impact cardiology journals may help to improve the burgeoning problem of lower citation rates among non-white vs. white scholars [122].

## 4. Conclusions

Achieving health equity in populations with congenital heart disease (CHD) throughout the lifespan is possible if the contributing factors begin to be addressed at the institutional and system levels. These interventions require a multipronged approach with a critical examination of multilevel factors and health policies that continue to drive health inequities. Minoritized populations with CHD who have fallen victim to structural racism and consequently suffer poorer SDOH deserve actionable strategies at the individual, institutional, and system levels to reduce health disparities.

## Data Availability

Data sharing not applicable, as no new data were created or analyzed in this study. Data sharing is not applicable to this article.
